# Setting an International Research Agenda for Fear of Cancer Recurrence: An Online Delphi Consensus Study

**DOI:** 10.3389/fpsyg.2021.596682

**Published:** 2021-02-22

**Authors:** Joanne Shaw, Helen Kamphuis, Louise Sharpe, Sophie Lebel, Allan Ben Smith, Nicholas Hulbert-Williams, Haryana Mary Dhillon, Phyllis Butow

**Affiliations:** ^1^Psycho-oncology Co-operative Research Group, Faculty of Science, School of Psychology, The University of Sydney, Sydney, NSW, Australia; ^2^Faculty of Science, School of Psychology, The University of Sydney, NSW, Australia; ^3^School of Psychology, University of Ottawa, Ottawa, ON, Canada; ^4^Centre for Oncology Education and Research Translation (CONCERT), South Western Sydney Clinical School, Ingham Institute for Applied Medical Research, University of New South Wales, Sydney, NSW, Australia; ^5^Centre for Contextual Behavioural Science, School of Psychology, University of Chester, Chester, United Kingdom; ^6^Faculty of Science, Centre for Medical Psychology and Evidence-based Decision-making, The University of Sydney, NSW, Australia

**Keywords:** cancer, fear of cancer recurrence, Delphi method, research priorities, international

## Abstract

**Background:**

Fear of cancer recurrence (FCR) is common amongst cancer survivors. There is rapidly growing research interest in FCR but a need to prioritize research to address the most pressing clinical issues and reduce duplication and fragmentation of effort. This study aimed to establish international consensus among clinical and academic FCR experts regarding priorities for FCR research.

**Methods:**

Members of the International Psycho-oncology Society (IPOS) Fear of Cancer Recurrence Special Interest Group (FORwards) were invited to participate in an online Delphi study. Research domains identified in Round 1 were presented and discussed at a focus group (Round 2) to consolidate the domains and items prior to presentation in further survey rounds (Round 3) aimed at gaining consensus on research priorities of international significance.

**Results:**

Thirty four research items were identified in Round 1 and 33 of the items were consolidated into six overarching themes through a focus group discussion with FCR experts. The 33 research items were presented in subsequent rounds of the delphi technique. Twenty one participants contributed to delphi round 1, 16 in round 2, and 25 and 29 participants for subsequent delphi rounds. Consensus was reached for 27 items in round 3.1. A further four research items were identified by panelists and included in round 3.2. After round 3.2, 35 individual research items were ratified by the panelists. Given the high levels of consensus and stability between rounds, no further rounds were conducted. Overall intervention research was considered the most important focus for FCR research. Panelists identified models of care that facilitate greater access to FCR treatment and evaluation of the effectiveness of FCR interventions in real world settings as the two research items of highest priority. Defining the mechanisms of action and active components across FCR/P interventions was the third highest priority identified.

**Conclusion:**

The findings of this study outline a research agenda for international FCR research. Intervention research to identify models of care that increase access to treatment are based on a flexible approach based on symptom severity and can be delivered within routine clinical care were identified as research areas to prioritize. Greater understanding of the active components and mechanisms of action of existing FCR interventions will facilitate increased tailoring of interventions to meet patient need.

## Introduction

Early diagnosis and improved treatments for cancer have led to higher survival rates (Arnold et al., 2019). With improved survival, there are increasing numbers of people living with the fear that their cancer will recur (Koch et al., 2013). New treatments have also led to increased numbers of people with advanced disease living with uncertainty (Thewes et al., 2017). While some degree of fear of recurrence (FCR) defined as “*fear, worry, or concern relating to the possibility that cancer will come back or progress”* (Lebel et al., 2016)(pg3267) is considered normal, nearly 50% of survivors experience moderate to high levels, suggestive of clinical FCR. High levels of FCR can persist over time (Crist and Grunfeld, 2013) and negatively impact psychological wellbeing and quality of life, resulting in increased utilization of healthcare resources (Lebel et al., 2013; Simard et al., 2013; Thewes et al., 2013). FCR is one of the most prevalent and severe unmet supportive care needs reported, with Simard et al. reporting in their review that up to 79% of cancer survivors reporting FCR as an unmet need or concern (Simard et al., 2013).

For these reasons, research interest in FCR has grown rapidly, evident from a doubling of the number of publications (based on a PubMed database search conducted on June 2020) from a mean publication rate of 57 per year (2010−2014) to 108 per year (2015−2020), including the publication of 12 systematic reviews (Crist and Grunfeld, 2013; Koch et al., 2013; Simard et al., 2013; Thewes et al., 2013; Ozga et al., 2015; Fardell et al., 2016; Simonelli et al., 2017; Yang et al., 2017a,b, 2019; Hall et al., 2018; Tauber et al., 2019). These reviews summarize a broad range of research activity including identification of factors associated with FCR (Crist and Grunfeld, 2013; Ozga et al., 2015), FCR measurement (Thewes et al., 2013), FCR interventions and management (Simonelli et al., 2017; Hall et al., 2018; Tauber et al., 2019), theoretical frameworks explaining FCR (Fardell et al., 2016) and prevalence, determinants and consequences of FCR (Simard et al., 2013; Yang et al., 2017a,b, 2019) as well as different care delivery models (Liu et al., 2019) and highlight that much of the research has been focused on FCR in breast cancer or mixed cancer samples composed mainly of colon, prostate, or lung and breast cancer patients (Simard et al., 2013). However, despite the increasing volume of FCR research and progress made in areas such as FCR treatment, fundamental questions regarding assessment, optimal intervention across patient groups, and determinants of severity are still to be answered (Sharpe et al., 2017). Establishing priorities for FCR research will increase the quality and clinical utility of findings, and reduce the potential for research waste through duplication and/or fragmentation of effort, and increased co-ordination of research will enhance funding competitiveness (Cristea and Naudet, 2019; Sullivan et al., 2019).

A recent priority-setting exercise for FCR research in Australia provided guidance for local research activities (Butow et al., 2019), however, these may not generalize more broadly given differences in settings delivering psycho-oncology care internationally. Similarly, although a review of ovarian cancer-specific psychosocial research gaps identified FCR as an important area for further research—the scope and focus of the FCR agenda for this patient group was not explored (Goarin et al., 2020). Priority setting exercises have demonstrated their potential to inform research. For example, a 2 day FCR colloquium in Ottawa, Canada (Lebel et al., 2017) not only provided a consensus definition for FCR but also highlighted the need for research to standardize measurement and development of theoretically informed interventions. Fostered through the establishment of a Special Interest Group under the banner of the International Psycho−Oncology Society (FORwards), these objectives have driven FCR research to date, but have now largely been met. In this study, we aimed to build on previous priority setting exercises and gain consensus on international FCR research priorities to foster collaborative research efforts and optimize FCR outcomes for adult cancer patients.

## Materials and Methods

### Study Design

A mixed methods approach was adopted to identify research priorities based on the methodology used in previous priority setting activities (Butow et al., 2019). Round 1 of the Delphi to identify research domains of potential importance, involved completion of an online survey. The survey results were presented and discussed at a focus group (Round 2) to consolidate the domains identified in round 1 prior to presentation in further survey rounds aimed at gaining consensus on research priorities of international significance to guide future collaborative research. The Delphi technique is a research methodology for establishing consensus among experts through a series of questionnaires (Keeney and McKenna, 2011) and commonly used to identify research priorities (Downing et al., 2015; Butow et al., 2019; Sullivan et al., 2019).

### Participants

Participants were recruited through the International Psycho-oncology Society (IPOS) fear of cancer recurrence special interest group (FORwards). IPOS is an international multidisciplinary professional network of researchers and clinicians (*n* = 598 from 68 countries) established to foster clinical and research collaborations to support the psychosocial health of those affected by cancer. The FORwards special interest group is comprised of IPOS members with an interest in FCR (*n* = 85 from 23 countries). The broad aims of the group are to raise awareness of FCR and promote activities to improve the identification and treatment of high levels of FCR amongst cancer patients and the group has a strong focus on stimulating multi-disciplinary, international FCR research. FORwards members were emailed an invitation to participate in each phase of the Delphi, however, for the focus group, it was a requirement that members be present at the IPOS World Congress, Banff Canada since this component required face to face participation, and Banff represented an ideal opportunity when a large number of FCR experts were already present. The study was approved by the University of Sydney human ethics committee (HREC 2019/608).

### Data Collection

#### Identification of Research Domains

##### Round 1

FORwards special interest group members were emailed an invitation to participate in round 1 of the Delphi—an online survey. Interested participants provided online consent prior to accessing the survey. The survey was constructed and administered using RedCap (Research Electronic Data Capture) database, a secure, web-based software platform designed to support data capture for research studies (Harris et al., 2009). The descriptive survey asked respondents to indicate what they perceived to be the three most important FCR research priorities of international significance. Survey responses were collected as free text variables and content analyzed using a conventional approach (Hsieh and Shannon, 2005) by two researchers independently (JS and PB). Any disagreements were resolved through discussion to ensure consensus. The analysis identified a list of individual items, which were grouped into higher-order themes to identify research domains. The individual items were weighted (score of 3 = first priority, 2 = second priority, 1 = third priority) and mean scores for each item were used to calculate an overall ranking of research priorities.

##### Round 2

The results of the survey were presented to FORwards members attending the IPOS World Congress in Banff Canada (September 2019) as part of a 90 min focus group convened specifically to review and discuss FCR research priorities. An experienced qualitative researcher (JS) conducted the focus group and two researchers documented the discussion. The results of the content analysis were presented to the focus group including each domain and associated items. The focus group followed a semi structured format with the facilitator guiding initial discussion and allowing participants to openly discuss potential research questions and priorities. All new research items generated through group discussion were included in the focus group transcript. Thematic analysis (Braun, 2012) of participant responses was conducted by two researchers independently (JS and PB) and an agreed coding scheme developed, with subsequent higher-order analysis to identify overarching themes. The transcripts were reviewed line-by-line by a single author (JS) who searched for concepts, themes, and ideas, and developed a preliminary coding scheme. Transcripts were read and coded by a second author independently (PB) who compared and discussed their individual coding choices. Any disagreements were resolved by discussion. The coding scheme was revised based on these discussions to develop a final coding structure. No *a priori* assumptions regarding priorities were made by the researchers. The researchers inductively developed two categories of descriptive themes from the data—the research priorities expressed by the participants and the rationale participants used to explain their research priorities.

#### Gaining Consensus for Research Priorities

##### Round 3

Research items generated through the item identification phase were presented to FORwards members using an online Delphi consensus methodology. Members were emailed an invitation to participate in an online consensus process and the survey was constructed and administered using RedCap. In each round, panelists indicated their agreement on the importance of each research item on a 5-point Likert scale (1 = very important to 5 = not important at all). Panelists were able to provide free text comments explaining the reasons for their rating. Consensus was defined *a priori* as 80% or more of panelists rating the item either important/very important or not important/not at all important. Panelists were also able to indicate if there were other research priorities they perceived as important, but not included.

Data from round 3.1 of the consensus process was summarized and any new items, as well as items where consensus was not reached (with percentages endorsing each response option and any free-text responses), were re-presented in a second round (i.e., round 3.2). We had an *a priori* stopping rule that no further rounds would be conducted when there were fewer than two items where consensus was achieved. In round 3.2, panelists also nominated their top three research priorities, ranking them from 1 (most important) to 3 (3rd most important). Non-responders were e-mailed up to three reminders for each round.

### Statistical Analyses

Data were exported from RedCap into an excel spreadsheet and analyzed descriptively. Percentage agreement was calculated based on the number of respondents who agreed/strongly agreed or disagreed/strongly disagreed on the importance an item, divided by the total number of respondents as a percentage. SPSS Statistics 24 (IBM, Armonk NY) was used to calculate mean importance for each item. Lower mean scores indicated higher perceived priority. From round 3.2 data, the top three ranked research priorities from the total list of research items was calculated. The individual items were weighted (score of 1 = first priority, 2 = second priority, 3 = third priority) and total scores for each item were used to calculate an overall ranking. Research domains were also ranked using Kendalls co-efficient W to assess ranking agreement among respondents.

## Results

### Round One: Identification of Research Domains

Twenty-one (25%) FORwards members completed the online survey. The majority of respondents were psychologists (66%, *n* = 14), with > 10 years working in oncology (71%, *n* = 15). Sixty six percent (*n* = 14) of respondents indicated research accounted for > 50% of their time, and 81% (*n* = 17) indicated they were currently involved in FCR research. The survey elicited 34 individual research items across five higher order themes to identify research domains: (1) intervention research, (2) screening and measurement, (3) model and/or guideline development, (4) etiology of FCR, and (5) implementation research. The full list of research items and associated weighting is provided as Supplementary Tables 1,2.

### Round Two: Consolidation of Research Items

Sixteen (19%) FORwards members (12 psychologists, two nurses and two psychology graduate students involved in FCR research) from nine countries participated in the focus group. Thematic analysis of the focus group discussion transcript resulted in refinement and reconceptualization of the research items identified from the survey and consolidation of 34 items across six overarching research domains: (1) intervention models (*n* = 8 items), (2) researching specific populations (*n* = 7 items), (3) definitions and mechanisms of action for FCR (*n* = 8 items), (4) description of FCR predictors and consequences (*n* = 4 items), (5) detection and screening (*n* = 3 items), and (6) implementation (*n* = 4 items). All 34 research items were presented as part of the Delphi process and are listed in Table 2.

### Round Three: Consensus for Research Items

#### Participants

Twenty five (29% response rate) FORwards members participated in round 3.1 of the Delphi consensus survey and 29 (34%) participated in round 3.2. The mostly female (83%) respondents were representative of the disciplines and international regions engaged in FCR research and IPOS membership (Table 1).

**TABLE 1:** Delphi round 3 respondent characteristics.

Variable		Round 3.1	Round 3.2	Proportion of
		(*n* = 25)	(*n* = 29)	IPOS
		N (%)	N (%)	membership**
Age	26−30	5 (20)	2 (7)	
	31−40	7 (28)	10 (36)	
	41−50	7 (28)	6 (21)	
	51−60	4 (16)	7 (25)	
	61−69	2 (8)	3 (11)	
	>70	0 (0)	0 (0)	
Gender	Female	20 (80)	24 (82)	478 (80)
	Male	5 (20)	5 (18)	120 (20)
Discipline	Psychology	14 (56)	17 (59)	244 (41)
	Psychiatry	1 (4)	2 (7)	80 (13)
	Nursing	1 (4)	2 (7)	40 (7)
	Social work	0 (0)	0 (0)	14 (1)
	Oncology	0 (0)	0 (0)	18 (2)
	Research	9 (36)	8 (27)	83 (14)
Country *	Australia	5 (20)		63 (11)
	Canada	4 (16)		30 (5)
	Denmark	1 (4)		7 (1)
	Japan	1 (4)		22 (4)
	Mexico	1 (4)		11 (2)
	Netherlands	3 (12)		11 (2)
	Portugal	1 (4)		2 (0.3)
	Russia	1 (4)		7 (1)
	South Korea	1 (4)		4 (0.7)
	Spain	2 (8)		3 (0.5)
	United Kingdom	2 (8)		25 (4)
	United States of America	3 (12)		93 (16)
Research role	Up to 5%	1 (4)	1 (3)	
	5−10%	3 (12)	2 (7)	
	10−15%	2 (8)	3 (11)	
	15−25%	0 (0)	0 (0)	
	25−50%	4 (16)	5 (17)	
	50−75%	4 (16)	5 (17)	
	75−100%	11(44)	13 (45)	

*Due to a programming error country was not collected in round 2. **Only limited demographic data collected by IPOS.

#### Consensus Rating

Thirty-three research items were initially presented to panelists and 27 of these reached consensus (≥80% agreement). Panelists also identified 4 additional items in round 3.1, including two items conceptualized under an additional domain *Uncertainty and avoidance.* The items where consensus was not reached (*n* = 7) and the additional four items were re-presented to panelists in round 3.2. Consensus was reached on nine items. Two items failed to reach consensus: *Explore the underlying similarities and differences between Fear of Cancer Recurrence (FCR) and Fear of Progression (FoP)* (72.4% agreement) and *Examine the effects of FCR/P on use of alternative therapies and their costs (69% agreement)*. Thus, these two items were eliminated. In summary, given the stability across rounds, 35 individual research items were ratified by the panelists and no further rounds were conducted. Table 2 lists individual items grouped by domain and final level of consensus for each item in ranking order.

**TABLE 2:** Mean ratings and level of consensus for fear of cancer recurrence international research priorities.

Research priority	Round 3.1		Round 3.2	
Intervention models	Mean (*SD*)*	% agreement (n)	Mean (*SD*)*	% agreement (n)
Develop and evaluate more accessible models (low cost, online, telehealth, group, blended models of care, non-mental health delivered)	1.32 (0.56)	96 (25)		
Evaluate the effectiveness of existing interventions in real world settings (pragmatic trials)	1.48 (0.77)	92 (25)		
Develop and evaluate brief FCR/P interventions (suitable for inpatients, single session interventions at follow up appointments)	1.6 (0.71)	88 (25)		
Develop and evaluate stepped care interventions for low, sub-clinical, clinical levels of FCR/P	1.56 (0.71)	88 (25)		
Develop and evaluate early interventions for prevention of FCR/P	1.64 (0.76)	84 (25)		
Develop international collaborations for FCR/P intervention studies and evaluate cross-cultural validity of interventions	1.6 (0.87)	84 (25)		
Develop and evaluate interventions for sub-clinical levels of FCR/P	2.12 (0.78)	72 (25)	1.86 (0.74)	86 (29)
Develop and evaluate FCR/P interventions delivered in community settings (non-cancer settings)	1.92 (0.81)	72 (25)	1.79 (1.01)	86 (29)
**Reaching specific populations**				
Explore FCR/P in “avoidant” people (i.e., those people who manage FCR/P by avoiding health-related behaviors/follow up appointments)	1.6 (0.65)	92 (25)		
Explore FCR/P in patients having new treatments (e.g., immunotherapy, targeted therapy, etc.) on FCR/P	1.76 (0.83)	84 (25)		
Explore FCR/P in caregivers of people with cancer (parents or relatives of people with cancer)	1.72 (0.84)	84 (25)		
Explore Fear of Progression (FoP) in patients with advanced disease (for whom existential issues are more relevant)	1.76 (0.97)	80 (25)		
Explore FCR/P in survivors of childhood cancers/adolescent and young adult cancer	1.72 (0.89)	80 (25)		
Explore the prevalence of FCR/P across cancer groups	2.2 (0.91)	56 (25)	1.66 (0.86)	90 (29)
Explore how caregivers and people with cancer influence each other’s FCR/P	not yet defined		1.52 (0.69)	86 (29)
**Definitions and mechanisms of action**				
Describe trajectories of FCR/P and identify covariates to explain any differences	1.64 (0.64)	92 (25)		
Map interventions to the different FCR/P trajectories	1.56 (0.65)	92 (25)		
Define the mechanisms of action and identify the active components across FCR/P interventions	1.28 (0.61)	92 (25)		
Refine the definitions of FCR and FoP	172 (0.89)	88 (25)		
Refine the theoretical model that explains FCR/P	1.60 (0.82)	88 (25)		
Explore the relationship between FCR/P and healthy anxiety	1.76 (0.72)	84 (25)		
Explore the underlying similarities and differences between Fear of Cancer Recurrence (FCR) and Fear of Progression (FoP)	1.88 (0.97)	76(25)	1.79 (1.01)	72 (29)
Explore the relationship between FCR/P and tolerating uncertainty more generally	1.76 (0.83)	72 (25)	1.31 (0.54)	96 (29)
**Description of FCR/P predictors and consequences**				
Examine the effects of FCR/P on healthcare seeking/health service utilization and associated costs	1.48 (0.59)	96 (25)		
Establish predictors of clinically significant FCR/P	1.48 (0.65)	92 (25)		
Establish predictive models for FCR/P	1.60 (0.76)	84 (25)		
Explore the impact of FCR/P on clinical outcomes (e.g., adherence to treatment)	1.64 (0.81)	80 (25)		
Examine the effects of FCR/P on use of alternative therapies and their costs	not yet defined		1.97 (0.98)	69 (29)
**Detection and screening**				
Develop better clinical and outcome measures of FCR/P which reflect clinical criteria for FCR/P	1.44 (0.65)	92 (25)		
Develop better FCR/P screening measures which reflect clinical criteria for FCR/P	1.36 (0.64)	92 (25)		
Establish clear clinical cut-offs for FCR/P screening measures (to guide intervention recommendations)	1.52 (0.71)	88 (25)		
**Implementation**				
Develop FCR/P clinical guidelines	1.50 (0.66)	92 (24)		
Develop and evaluate implementation research outcomes for FCR/P interventions	1.52 (0.79)	83 (23)		
Conduct health economic analyses of FCR/P intervention	1.83 (0.92)	75 (24)	1.24 (0.44)	100 (29)
Evaluate implementation of routine screening for FCR/P	1.78 (0.85)	74 (23)	1.24 (0.51)	97 (29)
**Uncertainty and avoidance**				
Explore the relationship between uncertainty and FCR/P	Not yet defined		1.59 (0.73)	86 (29)
Explore the relationship between avoidance, uncertainty and FCR/P	Not yet defined		1.59 (0.78)	83 (29)

*Mean of reported scores for each item where 1 = very important to 5 = not at all important (possible range 1−5).

### Research Priorities Ranking

The mean ranking of the seven research domains is listed in Table 3. Lower mean score is indicative of higher ranking of importance. Although all the priorities and related items were deemed important, *Intervention models* was identified as the highest priority (rank 3.04) and *Uncertainty and Avoidance* lowest (rank 5.04). In line with the ranked research domains, when asked to indicate their top three research items in order of importance, participants nominated two research items related to intervention models—*Develop and evaluate more accessible models* and *Evaluate the effectiveness of existing interventions in real world settings*, followed by *Define the mechanisms of action and identify the active components across FCR/P interventions* as the research of highest priority. Individual research items listed by >10% of participants as one of their top three priorities are listed in Table 4. Of note, although the item *Explore the underlying similarities and differences between Fear of Cancer Recurrence (FCR) and Fear of Progression (FoP)* failed to gain consensus, five panelists ranked this item as their 2nd or 3rd most important research domain.

**TABLE 3:** Mean rank of overall research themes.

Research themes	Mean rank (Kendalls W)*
Intervention models	3.04
Definitions and Mechanisms of Action for FCR/P	3.25
Detection and Screening	3.64
Description of FCR/P predictors and consequences	4.00
Implementation	4.36
Reaching specific populations	4.68
Uncertainty and Avoidance	5.04

*Lower mean scores = higher perceived priority.

**TABLE 4:** Top three ranking for individual research topics across research themes *.

Item	Priority 1 (n)	Priority 2 (n)	Priority 3 (n)	Total No participants rating priority 1−3
Develop and evaluate more accessible models—low cost, online, telehealth, group, blended models of care, non-mental health delivered (theme 1)	6	3	5	14 (48%)
Evaluate the effectiveness of existing interventions in real world settings (theme 1)	5	1	3	9 (31%)
Define the mechanisms of action and identify the active components across FCR/P interventions (theme 3)	3	1	2	6 (20.6%)
Explore the relationship between avoidance, uncertainty, and FCR/P (theme 7)	3	0	2	5 (17.2%)
Develop and evaluate stepped care interventions for low, sub-clinical, clinical levels of FCR/P (theme 1)	2	3	0	5 (17.2%)
Explore the underlying similarities and differences between Fear of Cancer Recurrence (FCR) and Fear of Progression (FoP) (theme3)	0	2	3	5 (17.2%)
Explore FCR/P in patients having new treatments (e.g., immunotherapy, targeted therapy, etc.) on FCR/P (theme2)	1	0	3	4 (13.8%)
Develop and evaluate early interventions for prevention of FCR/P (theme1)	1	2	1	4 (13.8%)
Refine the theoretical model that explains FCR/P	0	1	3	4 (13.8%)

**^∗^Only topics listed by > 10% of participants are reported.**

## Discussion

Through the use of Delphi methods, this study developed consensus among key clinical and research stakeholders regarding aspects of FCR research that should be prioritized in future international collaborations. Based on the overall ranking of overarching themes, development and evaluation of intervention models was considered the most important focus for FCR research. In line with this, stakeholders identified development and evaluation of models of care that facilitate a greater access to FCR treatment and evaluation of the effectiveness of FCR interventions in real world settings as the two research questions of highest priority, with high levels of consensus (96 and 92%, respectively). Stakeholders also highlighted the importance of further work related to defining the mechanisms of action and active components across FCR/P interventions, ranking this research as the third highest priority.

The results of this priority setting exercise confirm that although the existing treatment protocols incorporating interventions delivered by specialist mental health clinicians are effective (Tauber et al., 2019), there remains a need to evaluate new service delivery models aimed at increasing accessibility. Preliminary research investigating use of online delivery and oncologist and nursing-led interventions reflects this move to more accessible alternatives (Liu et al., 2019; Cruickshank et al., 2020; Reb et al., 2020; Smith et al., 2020; Wagner et al., 2020). The focus on flexible delivery options is not surprising given increasing international efforts directed toward survivorship care models better tailored to address the needs-support gap (Carolan et al., 2018). For cancer survivors dealing with FCR, evidence-based models of care outside of the hospital-setting are perceived as a valid means of reducing the burden of distress experienced (Smith et al., 2020), although these interventions have yet to demonstrate their efficacy (van Helmondt et al., 2020).

Given FCR treatment models are currently time and resource intensive, there was also a clear support for evaluation of stepped care approaches incorporating interventions tailored to FCR severity, such that more intensive interventions (psychologist delivered face to face sessions) are reserved for patients with clinical levels of FCR who do not benefit from simpler first line approaches (online or non-mental health delivered). A stepped care approach is, however, contingent on the identification of clinical cut-offs to appropriately identify the intensity of intervention best suited to the severity of symptoms experienced. The stepped care approach to anxiety and depression screening and management as outlined in the clinical pathway for identification and management of anxiety and depression in cancer (Butow et al., 2015) provides a useful framework for the development of a multi-disciplinary evidence-based guidance for FCR management. Although trials of evidence-based stepped care models have not yet been reported, a number of brief interventions among patients with low levels of FCR and interventions aimed at FCR prevention hold promise for this approach (Davidson et al., 2018; McHale et al., 2020). Prevention of FCR was also highlighted as a key area for future research. Finally, stakeholders acknowledged that efficacy of FCR interventions has been confirmed under ideal settings within randomized controlled trials targeting early stage, primarily breast cancer patients (Simonelli et al., 2017). To facilitate a greater translation of evidence into practice, they highlighted the need to conduct pragmatic trials, encompassing more diverse patient groups both in terms of disease and socio-demographic factors, conducted in routine clinical settings that reflect a diversity of cultural contexts, to ensure interventions are evaluated under real world conditions (Sanson-Fisher et al., 2019).

Despite the dissemination of a number of FCR conceptual models (Lee-Jones et al., 1997; Mellon et al., 2007; Fardell et al., 2016; Simonelli et al., 2017; Lebel et al., 2018) and exploration of relationships between FCR and related constructs [e.g., death anxiety (Sharpe et al., 2018), pain (Heathcote and Eccleston, 2017)], stakeholders reported research to better understand the mechanisms of action and active components of FCR interventions as one of their top three priorities. A number of recent studies have reported moderators and mediators of FCR in terms of patient level characteristics (Herschbach et al., 2010; Smith et al., 2018) and mechanisms of treatment efficacy (Lebel et al., 2018; Sharpe et al., 2019). A meta-analysis of FCR interventions also found that more contemporary forms of CBT aimed at modifying thought *processes* (i.e., how individuals relate to their inner experiences, rather than thought *contents*) were more effective (Simonelli et al., 2017). However, the link between cognitions and behaviors common in FCR, and individual intervention components, has not been established. Conducting comparative effectiveness studies (i.e., head to head trials) between FCR interventions may also help to better understand the relative merits of different interventions (Hulbert-Williams et al., 2018). Stakeholders confirmed such information would facilitate not only replication of findings, but also inform optimization of existing interventions in terms of treatment dose and determining where active elements can be adapted or modified in real-world settings (Rankin et al., 2019).

Understanding the mechanisms of action can also help to predict who might best benefit from available treatments. Stakeholders questioned whether the differing prognostic outcomes of disease influence the FCR trajectory for individual patients in clinically meaningful ways. Ratings indicated that stakeholders perceived that exploring potential differences across prognostic categories will inform development of interventions that better target recurrence and progression of fears across different patient groups.

An interesting finding of this study, which differs from the findings of the Australian Delphi study (Butow et al., 2019), was the support for greater research to explore the relationship between avoidance, uncertainty, and FCR. Stakeholders noted that while current conceptualizations of FCR have focused on those cognitions that drive hypervigilance, less attention has been given to patients who engage in avoidant behaviors. This patient group was of particular concern to the stakeholders as the risk of recurrence was higher due to their avoidant health behaviors aimed at reducing fear and uncertainty (Simonelli et al., 2017). The role of uncertainty, which has previously been linked with increased FCR (Lebel et al., 2018), was also highlighted as an important area of research in light of newer immune and targeted therapies which offer uncertain futures to patients with advanced disease (Levy et al., 2019).

Finally, it should be noted that two research topics proposed in the initial survey and confirmed during the focus group discussion failed to reach consensus. However both topics were conceptually similar to other items within the Delphi where consensus was reached; for example, the item *explore the underlying similarities and differences between FCR and fear of progression* (72.4% agreement) is closely related to a number of other items within the Definitions and Mechanisms of Action theme such as refining the *definitions of FCR and FoP* and the theoretical model *that explains FCR/P* both of which reached consensus (88%). Similarly, the item *effects of FCR/P on use of alternative therapies and their costs* (69% agreement) may have been subsumed into the broader item *examine the effects of FCR/P on healthcare seeking/health service utilization and associated costs* (96% agreement).

The results of this consensus study need to be viewed in light of a number of limitations. Firstly, although all members of the FORwards special interest group were invited to participate at each phase of the research, overall participation rates were low with less than one third of members completing the Delphi study. Additionally, FORwards members are predominately from Western-culture based and OECD nations, limiting potential multicultural considerations of FCR research. Participation in the focus group was limited to only those FORwards members able to attend the IPOS World Congress. Despite the low recruitment rate, those who did participate represented key opinion leaders in the field and therefore were able to provide expert advice on the state of current international research. There was also there was limited representation of stakeholders from developing nations, although given the limited psycho-oncology services available, the findings around the need for increased FCR treatment accessibility may not have changed. A strength of this study was the international multidisciplinary participation, although there was an over-representation of psychologists. However, given that psychologists are the discipline most commonly treating FCR, their engagement with this agenda setting supports the clinical relevance of recommendations resulting from the consensus process. Finally, although the conduct of the Delphi was based on best practice methodologies, the majority of research items were rated highly, and once consensus was reached were not re-presented in subsequent rounds. Re-presenting items after consensus was reached may have provided greater differentiation of research priorities and better refined some of the lower ranked priorities. However, the high levels of consensus across a broad range of themes highlights the breadth of research questions yet to be answered. Given stakeholders were able to identify the three most important research topics to be addressed internationally and are largely consistent with the priorities identified by the Australian Delphi study (Butow et al., 2019), despite the study limitations, the findings confirm a clear direction to researchers and funding bodies about the nature of the research that should be considered in the immediate future.

## Conclusion

The findings of this study provide a roadmap for international FCR research. Stakeholders confirmed intervention research to identify models of care that increase access to treatment, are based on a stepped care approach, and are able to deliver as part of routine clinical care as the highest priorities for future research. Greater understanding of the active components and mechanisms of action of existing FCR interventions will also facilitate greater tailoring of interventions to meet patient need.

## Data Availability Statement

The raw data supporting the conclusions of this article will be made available by the authors, without undue reservation.

## Ethics Statement

The studies involving human participants were reviewed and approved by the University of Sydney Human Research Ethics Committee (2019/608). The patients/participants provided their written informed consent to participate in this study.

## Author Contributions

JS lead the data collection and analysis. PB and HK contributed collection and analysis. JS lead manuscript development. All authors contributed to review and finalization of the manuscript, study conceptualization and design, and interpretation of results.

## Conflict of Interest

The authors declare that the research was conducted in the absence of any commercial or financial relationships that could be construed as a potential conflict of interest.
