# Supplementary material for: Setting an International Research Agenda for Fear of Cancer Recurrence: An Online Delphi Consensus Study
Source: Front Psychol. 2021 Feb 22;12:596682. doi: 10.3389/fpsyg.2021.596682 (PMC7938308; doi:10.3389/fpsyg.2021.596682)
Supplement: Supplementary file 1 [file Table_1.docx]

**Supplementary Tables**

Supplementary Table 1: Survey Sample Characteristics

|  | **Participants n (%)** |
| --- | --- |
| **Discipline** |  |
| Psychology | 14 (66) |
| Psychiatry | 1 (4.7) |
| Nursing | 2 (9.5) |
| Public health | 1 (4.7) |
| Other (researcher) | 3 (14.3) |
| **Years in Oncology** |  |
| 1-2 | 2 (9.5) |
| 3-5 | 1 (4.7) |
| 6-10 | 3 (14.3) |
| >10 | 15 (71.4) |
| **Research Time (%)** |  |
| <25 | 4 (19) |
| 26-50 | 3 (14.3) |
| 51-75 | 7 (33) |
| 75-100 | 7 (33) |
| **FCR/P Research Participation** |  |
| yes | 17 (80.9) |
| no | 4 (19) |

Supplementary Table 2: Weighted Priorities: Survey

| **Individual Priority Items** | **Research Topic** | **Priority 1 Weighting** | **Priority 1 Weighting** | **Priority 1 Weighting** | **Total Weighted score** |
| --- | --- | --- | --- | --- | --- |
| Increased accessibility of interventions/low cost/online/non-mental health delivery | Intervention Research | 3 | 2 | 1 | 6 |
| Development of FCR/FoP screening tools | Screening/  measurement | 3 | 2 | 1 | 6 |
| Prevention of FCR | Intervention Research | 3 | 2 |  | 5 |
| Differences in FCR and FoP between early advanced cancer patients | FCR/P Etiology | 3 | 2 |  | 5 |
| Prevalence of FCR and FoP across cancer groups | FCR/P Etiology | 3 |  | 1 | 4 |
| Natural course of FCR/FoP over time and identification of covariates | FCR/P Etiology | 3 |  | 1 | 4 |
| Development of FCR clinical guidelines | Model/  guideline development | 3 |  | 1 | 4 |
| New structured or semi-structured behavioral and/or cognitive interventions to reduce FCR | Intervention Research | 3 |  |  | 3 |
| Short interventions suitable for: short term interventions, e.g. hospitalized patients in acute care, single-session interventions at follow-up appointments. | Intervention Research | 3 |  |  | 3 |
| Development of an FCR/FoP measure that captures clinical FCR | Screening/  measurement | 3 |  |  | 3 |
| Understanding of mechanisms of action for interventions | Model/  guideline development |  | 2 | 1 | 3 |
| Differences between FCR and FoP | FCR/P Etiology | 3 |  |  | 3 |
| Difference in FCR/FoP in patients with advanced cancer on ongoing treatment | FCR/P Etiology | 3 |  |  | 3 |
| Conduct components analysis of FCR interventions to facilitate tailoring | Implementation Research |  | 2 | 1 | 3 |
| Implementation of routine FCR screening | Implementation Research |  | 2 |  | 2 |
| Cost effectiveness analyses | Implementation Research |  | 2 |  | 2 |
| Implementation of interventions | Implementation Research |  | 2 |  | 2 |
| Identification of risk factors predictive of FCR | FCR/P Etiology |  | 2 |  | 2 |
| Cultural differences in FCR | FCR/P Etiology |  | 2 |  | 2 |
| FCR in advanced cancer | FCR/P Etiology |  | 2 |  | 2 |
| Stepped care models | Intervention Research |  | 2 |  | 2 |
| Investigate the relationship between FCR and health anxiety | Model/  guideline development |  | 2 |  | 2 |
| Theoretical development of FCR and measurement | Model/  guideline development |  | 2 |  | 2 |
| Identification of clinical FCR/FoP and inclusion in DSM/ICD | Model/  guideline development |  | 2 |  | 2 |
| Early intervention – online/group interventions | Intervention Research |  |  | 1 | 1 |
| Interventions for advanced disease | Intervention Research |  |  | 1 | 1 |
| Interventions for cultural minorities | Intervention Research |  |  | 1 | 1 |
| International collaborative studies | Intervention Research |  |  | 1 | 1 |
| Establishing clear cut-offs for different levels of FCR and defining the need for interventions per level | Screening/  measurement |  |  | 1 | 1 |
| Impact of new treatments on FCR/FoP | Screening/  measurement |  |  | 1 | 1 |
| Examining distal effects of fear of recurrence (e.g. health-care seeking/health care utilization, health behaviors) | Model/  guideline development |  |  | 1 | 1 |
| Comprehensive model of factors that impact on FCR | Model/  guideline development |  |  | 1 | 1 |
| Impact of FCR/FoP on clinical outcomes | FCR/P Etiology |  |  | 1 | 1 |
| Community models | Implementation Research |  |  | 1 | 1 |
